# Pooling as a strategy for the timely diagnosis of soil-transmitted helminths in stool: value and reproducibility

**DOI:** 10.1186/s13071-019-3693-3

**Published:** 2019-09-16

**Authors:** Marina Papaiakovou, James Wright, Nils Pilotte, Darren Chooneea, Fabian Schär, James E. Truscott, Julia C. Dunn, Iain Gardiner, Judd L. Walson, Steven A. Williams, D. Timothy J. Littlewood

**Affiliations:** 1 Department of Life Sciences, Natural History Museum, London, UK; 2 Department of Biological Sciences, Smith College, Northampton, MA USA; 3 Department of Infectious Disease Epidemiology, School of Public Health, Faculty of Medicine, Imperial College London, London, UK; 4 The Hospital for Sick Children, Toronto, ON Canada; 5 Molecular and Cellular Biology Programme, University of Massachusetts, Amherst, MA USA; 6 Core Research Laboratories, Natural History Museum, London, UK; 7 DeWorm3, Natural History Museum, London, UK; 8 Department of Global Health, University of Washington, Seattle, WA USA

**Keywords:** Breakpoint of transmission, Cost analysis of pooling, Pooling, Soil-transmitted helminths, Stool samples, qPCR-based diagnostics

## Abstract

**Background:**

The strategy of pooling stool specimens has been extensively used in the field of parasitology in order to facilitate the screening of large numbers of samples whilst minimizing the prohibitive cost of single sample analysis. The aim of this study was to develop a standardized reproducible pooling protocol for stool samples, validated between two different laboratories, without jeopardizing the sensitivity of the quantitative polymerase chain reaction (qPCR) assays employed for the detection of soil-transmitted helminths (STHs). Two distinct experimental phases were recruited. First, the sensitivity and specificity of the established protocol was assessed by real-time PCR for each one of the STHs. Secondly, agreement and reproducibility of the protocol between the two different laboratories were tested. The need for multiple stool sampling to avoid false negative results was also assessed. Finally, a cost exercise was conducted which included labour cost in low- and high-wage settings, consumable cost, prevalence of a single STH species, and a simple distribution pattern of the positive samples in pools to estimate time and money savings suggested by the strategy.

**Results:**

The sensitivity of the pooling method was variable among the STH species but consistent between the two laboratories. Estimates of specificity indicate a ‘pooling approach’ can yield a low frequency of ‘missed’ infections. There were no significant differences regarding the execution of the protocol and the subsequent STH detection between the two laboratories, which suggests in most cases the protocol is reproducible by adequately trained staff. Finally, given the high degree of agreement, there appears to be little or no need for multiple sampling of either individuals or pools.

**Conclusions:**

Our results suggest that the pooling protocol developed herein is a robust and efficient strategy for the detection of STHs in ‘pools-of-five’. There is notable complexity of the pool preparation to ensure even distribution of helminth DNA throughout. Therefore, at a given setting, cost of labour among other logistical and epidemiological factors, is the more concerning and determining factor when choosing pooling strategies, rather than losing sensitivity and/or specificity of the molecular assay or the method.

## Background

Pooling of faeces [1–5], urine [6, 7], serum [8] or disease vectors [9] have all been used as a cost-effective strategy to screen for infection present in the given substrate/matrix. Such an approach has been shown to provide accurate results, while reducing time and labour requirements. Additionally, but perhaps more so in the veterinary world than in any clinical mass drug administration (MDA) programme, ‘pooling’ as a strategy may allow for a rapid estimation of drug efficacy or infection prevalence present in the herd based on microscopy results and subsequent faecal egg counts (FECs) [10–13].

As previous goals to reduce the intestinal worm burden and morbidity in school-aged children have been extended and enriched with new programmes to achieve universal coverage of at-risk populations by 2030, new monitoring methods need to be implemented. Novel, precise and robust diagnostic tools that measure prevalence reduction and detect interruption of transmission are key to enable de-implementation of MDA programmes [14, 15]. Soil-transmitted helminths transmitted *via* the faecal-oral route (*Ascaris lumbricoides*, *Trichuris trichiura*, *Necator americanus*, *Ancylostoma duodenale*, *An. ceylanicum* and *Strongyloides stercoralis*) and/or *via* skin penetration (*N. americanus*, *An. duodenale*, *An. ceylanicum* and *S. stercoralis*) are amongst the neglected tropical pathogens drawing increased attention as targets for transmission interruption and possible elimination. Even though preventable, they affect almost a third of the world’s population [16]. However, surveillance of ongoing MDA programmatic efforts that aim to reduce the worm burden include thousands or tens of thousands of samples to be screened and analysed for STH-related prevalence, especially in low-prevalence areas where large sample sizes are required to accurately detect changes in infection. Previous attempts to evaluate pooling as a means of scaling soil-transmitted helminth diagnosis have yielded poor results. Such studies have relied upon microscopy as the diagnostic strategy [13, 17, 18], which lacks the sensitivity of molecular tools, such as quantitative polymerase chain reaction (qPCR); caveats and disadvantages of this approach have been thoroughly described previously [19, 20].

Such tools would ideally retain their sensitivity when samples from multiple individuals are combined, whilst minimizing the reagent cost implicated. More recent studies report additional cost granularity, including operational and logistical costs, concluding that a ‘pooling approach’ might not be as worthwhile as hoped [5]. These studies, however, have neither taken into account predicted pool sizes as optimal nor have they incorporated an adequately sensitive diagnostic tool; thus, such conclusions are yet to be confirmed. Modelling studies followed by experimental validations have suggested an optimal pooled sample range where pooling tends to be more cost-effective, whilst maintaining robustness and precision with minimal variation [12] but the decision whether to proceed with pooling or not will likely be based on a number of additional factors. Cost (determined by reagents, labour required, logistical and operational considerations), time (sample transportation and pool preparation) and the need for a sensitive enough diagnostic tool are not the only determinants which must be considered when deciding in favour, or in opposition, of pooling. The sample size of the study (*n*) and existing STH prevalence may also influence decision making [21].

Quantitative PCR has emerged as an effective molecular diagnostic tool to fill the need of heightened sensitivity compared to microscopy when infection levels drop considerably. Some of the advantages of qPCR include the theoretical ability to detect single numbers of eggs present in the faeces due to its analytical sensitivity, to distinguish between species [22, 23] and to achieve accurate results rapidly. Given these factors, qPCR may be the most likely currently available method to enable STH detection in pools in low-prevalence areas, especially when prevalence is close to the breakpoint of transmission [24]. For this reason, the use of PCR as part of a viable pooling strategy should be evaluated [25].

In settings with low-intensity of infections, the majority of samples screened are expected to be negative [26]. The sensitivity of a given method might increase or decrease when pooling is recruited; *increasing*, when multiple ‘weak’ infections are combined in a single pool, so collectively the target of interest is detectable by qPCR and *decreasing*, when a single infected sample is ‘buried’ among uninfected ones, and subsequently diluted, hence undetectable by qPCR [11].

A need for ‘spin-outs’ (subsequent tests) after testing the pools and the identification of the STH infection at an individual level may increase the cost of the ‘pooling approach’ substantially if required too often. This negates any advantages of the approach. Also, the risk of contamination is higher as testing larger pools of samples extends the handling and processing period and increases the risk for contamination, leading to false positive results, thus driving the cost higher, especially when re-extractions are needed to confirm individual infections [27]. When the sensitivity of an STH assay is decreasing, a very ‘weak’ infection might be missed in a pool of negatives. This could reduce the cost since collectively that pool would identify as negative so no added labour (or cost) for ‘spin-outs’ would be needed. As mentioned, any pool sizes higher than between 5 and 8 increases cost and time to prepare the pools and requires additional equipment.

Building on preliminary unpublished data gathered by members of our group, and taking into account the pool sizing predictive models, we examined the recruitment of pools of 5 as a tool for screening samples with low STH infection levels, aiming not to compromise either sensitivity or specificity of the qPCR. Additionally, the reproducibility of the protocol and agreement in two different laboratory settings was interrogated, and the necessity for multiple replicates obtained from each pool or individual samples was also evaluated. A basic cost exercise was performed through direct comparison of processing samples individually or as parts of pools. Also, without any prior knowledge regarding the distribution of the positive samples in a screened population, two scenarios were included in the cost-analysis based on different prevalence levels given; a ‘best-’ and a ‘worst-case’ scenario. Acknowledging that this analysis does not represent a mathematical cost model, we accounted simply for prevalence in a given sample population, labour time based on wages in different income settings and consumable costs based on standard list prices. Our results show that choosing whether to ‘pool or not to pool’ can only be determined effectively after considerable scrutiny of each of the component processes, which may be more problematic or prohibitory than loss of granular sensitivity of the diagnostic method used to detect the target of choice. Each process component should be taken into consideration before deciding in favour of pooling strategies.

## Methods

### Study design (phases I and II)

During phase I (‘seeding’ experiment) a series (*n* = 20) of infection-naïve stool samples purchased commercially (BioIVT; Westbury, NY, USA) were spiked with known numbers of *N. americanus* eggs mimicking low levels of infection as classified by the World Health Organisation (WHO) guidelines [28] and were mixed with four additional infection-naïve samples of equal volume to create pools of 5.

During phase II (field-samples experiment) of the study, aliquots from a series of field samples with known STH infection status, collected as part of an unrelated study, were mixed with four additional field samples (of equal volume) that had been tested and verified to be negative for all the five STH species of interest (see ‘strategic pooling’) to also create pools of five.

DNA extractions performed during phase I, and part of phase II, were conducted in different laboratories by different technicians to explore reproducibility of the developed protocol. Individual component samples were extracted alongside their pools throughout the process, and all extractions of both individual samples and pools were performed in duplicate (i.e. 1A, 1B, P1A and P1B). DNA from each pool was also extracted twice (PA_1&2_ and PB_1&2_). The sensitivity and specificity of the established protocol was evaluated by real-time PCR for each particular target helminth, and by all STH assays for the samples previously identified as negatives. Reproducibility of the protocol’s performance and agreement of results between the two different laboratories were also analysed.

### Phase I: ‘seeding’ experiment—Smith College (SC)

**Fig. 1:**
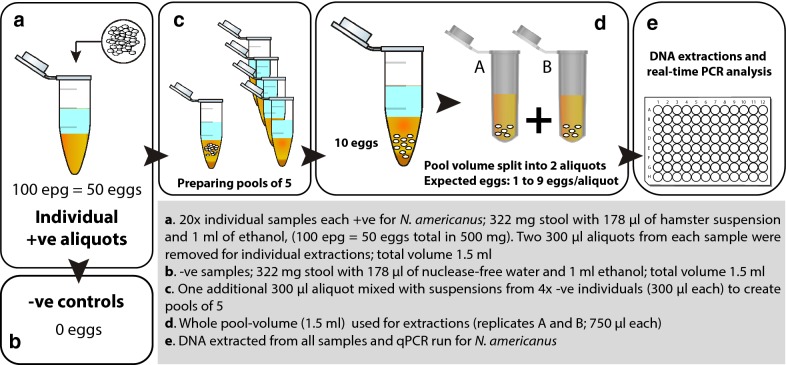
Schematic representation of the ‘seeding’ experiment (as proof of concept). Known egg counts of *N. americanus* eggs (in suspension) were utilized in order to spike individual, fixed volumes of naïve stool prior to mixing with four additional naïve stool aliquots of the same volume to form the pools of five

For use during ‘seeding’ experiments, performed at the Smith College (SC; Northampton, MA, USA), a suspension of hookworm eggs, utilized to spike the infection-naïve stool, was prepared as previously described [29]. In brief, hamster stool pellets with known infection levels expressed as eggs per gram (epg) were diluted in nuclease-free water such that 178 µl contained 50 eggs for a final infection-load of 100 epg (50 eggs in 500 mg of stool) (Fig. 1). The level of hookworm infection chosen was based on preliminary experiments where medium and high hookworm infection loads (based on WHO guidelines [28]) were employed, but showed abundancy of the target and early amplification detected by qPCR [30]; a primary concern of pooling is loss of sensitivity through dilution in low infection settings, so we chose a moderately low final concentration of 100 epg to detect potential dilution effects.

### Phase II: field-samples experiment—SC and Natural History Museum (NHM)

**Fig. 2:**
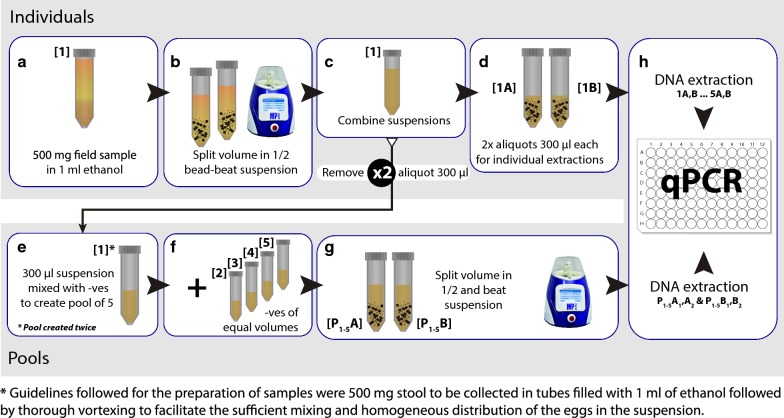
Schematic representation of the field-samples experiment. Previously screened faecal samples positive for one or more soil-transmitted helminths (STHs) were combined with four additional samples (of equal volume) identified as negative for all STHs to create pools of five (individual samples identified as negatives were also included in the study, as contamination controls). The DNA from every individual sample was extracted twice, each pool was formed twice and the DNA from each pool was also extracted twice. All the samples underwent qPCR for the target STH

At SC, a 34-sample panel was created for use in a proof-of-concept study. Thirty of these samples were positive for a single helminth (*A. lumbricoides*, *T. trichiura*, *An. ceylanicum*, *S. stercoralis*) and the remaining four were identified as negative. The volume of each sample (1.5 ml; 500 mg of stool suspended in 1 ml of ethanol) was split, homogenised and mixed with four infection-naïve stool aliquots of equal volume (Fig. 2). Another panel of 150 samples of human stool extracts, variously infected with the same species of STH (at least 500 mg of stool), was prepared at SC and was shipped to the Natural History Museum (NHM; London, UK). All samples utilized during phase II of this study were collected in Bangladesh as part of the WASH Benefits Bangladesh trial [31]. All samples were previously screened at SC *via* real-time PCR and the results for each individual sample were available. Amongst these samples, 130 were identified as negative for all species (*N. americanus*, *T. trichiura*, *A. lumbricoides*, *An. duodenale*, *An. ceylanicum* and *S. stercoralis*). The rest of the samples (*n* = 20) were identified as positive for at least one STH, with low/moderate intensity infections reported based on Kato-Katz/individual PCR data. For the generation of each positive pool, one sample identified as positive for at least one species of STH was mixed with four samples identified as negative. For the generation of negative pools, equal volumes of five negative samples were mixed (Fig. 2).

### Pool formation and DNA extraction

The total volume of each sample (1.5 ml stool in suspension) was divided into two aliquots and was homogenized using a high-speed bead beater (Fast Prep 5G, MP Biomedicals; Santa Ana, CA, USA) with Lysing Matrix E tubes (containing silica, glass bead and ceramic particles). The homogeneous suspensions were recombined into a single tube after the first lysis. Two ~ 300 µl aliquots of the suspension were transferred into two new Lysing Matrix E tubes for individual extractions (A and B) and two additional 300 µl were transferred to separate tubes designated for use in the constitution of pools (PA and PB). The same procedure was followed for all five samples that would form a single pool. After a pool was formed, the volume was split again, and a second homogenization following the same procedure occurred (second lysis). Following the second lysis step, two aliquots (300 µl each) from the pool (PA_1&2_ and PB_1&2_) were also subjected to DNA extraction. For all pools and individual samples, the same DNA extraction protocol was followed. All extractions began with an additional bead-beating step (the second homogenization step for individual samples and the third homogenization step for pooled samples). Extractions were then completed using the MP Bio Fast DNA SPIN kit for Soil (MP Biomedicals; Santa Ana, CA, USA) as previously described [29] (Figs. 1, 2). Following extraction, all samples were stored at – 20 °C until analysed *via* real-time PCR.

### Real-time PCR analysis

The cycling conditions, information on sequences from primers and probes and master mix used have all been previously described [22, 23, 29].

### Data and statistical analysis

To assess the diagnostic performance of the 5-sample pools, we calculated sensitivity, specificity, negative predictive value (NPV) and positive predictive value (PPV) in Excel v. 2016. Accuracy of the pooling method was also calculated using the formula: (true positives + true negatives)/number of pools. Confidence intervals (CI) for sensitivity, specificity, PPV and NPV were calculated using the Clopper–Pearson exact binomial method [32]. For these calculations qPCR results for the *individual* aliquots were considered as the ‘gold-standard’. Results for NHM and SC were calculated and presented separately and stratified by helminth species. Chi-square tests were conducted to determine whether there was statistical evidence of a difference in the sensitivity and specificity estimates between the two laboratories. To better understand how pooling impacted the (delayed) detection of the target compared to the individuals, Pearson’s correlation coefficient was used to quantitate the relationship between the qPCR outcome of the individual sample and that of the pooled one.

To investigate whether multiple extractions are required for each individual aliquot and/or 5-sample pool, Cohen’s kappa statistic [33] was calculated. This determines the degree of agreement in qPCR results (positive/negative) between A/B aliquots and between the 5-sample pool duplicates (PA_1_ and A_2_, PB_1_ and B_2_). Finally, for direct demonstration of agreement between the results obtained at NHM for the individual extracts and the ones originally screened as part of the independent study at SC (Bangladesh, WASH Benefits Bangladesh trial, see above), Cohen’s kappa statistic was also calculated.

### Cost exercise computation

Costs based on 1000 samples requiring processing (individually or as part of 5-sample pools) were calculated; the sample size was small enough for easy analysis and large enough to represent a case where pooling might be justified. For consistency and accurate reporting, the present protocol included all the extractions in duplicate and the formation and subsequent extraction of the same pool twice; these components were also part of the cost model and comparison. This cost exercise included labour and consumable costs (for plasticware and reagents per sample per assay run, based on list prices), tailored to a theoretically optimized version of the developed protocol (i.e. a protocol that would not process individual samples along with the pools simultaneously), as mentioned earlier.

Two separate case-scenarios were plotted for this exercise. In the simple case scenario, all the individual samples are negative (thus, so are the pools), and there is no need for ‘spin outs’; hence, only costs for labour and consumables (based on list prices online) are included. As part of a more complicated scenario, two different prevalence rates—with a single STH present for simplicity—were factored in; 2% which reflects the defined transmission breakpoint, and 15% as an indicator of prevalence when control programmes are needed and when pooling could be considered above individual sampling. In a ‘best-case’ complicated scenario, all positive samples would cluster together (e.g. 5 positive samples in a 5-sample pool). Whereas, in a ‘worst-case’ complicated scenario only one positive sample would be part of a 5-sample pool (e.g. mixed with four ‘negatives’).

## Results

Pooling was evaluated in terms of consistency, robustness, reproducibility and cost-effectiveness with comparisons made against individual sample results and between replicate pools.

Sensitivity of the 5-sample pooling technique differed between helminth species for both the samples tested at NHM and SC. *T. trichiura* had the lowest sensitivity for both NHM (0.65, 95% CI: 0.50–0.79) and SC (0.80, 95% CI: 0.64–0.91). All other helminth species from SC had absolute sensitivity (1.00, 95% CI: 0.40–1.00) whilst for NHM the highest sensitivity was obtained for *An. ceylanicum* (0.82, 95% CI: 0.60–0.95). For *T. trichiura* and *S. stercoralis* there was no evidence of a difference in sensitivity between NHM and SC (*P* = 0.13 and *P* = 0.22, respectively), whilst for *An. ceylanicum* there was weak evidence of a difference (*P* = 0.07) and for *A. lumbricoides* there was very strong evidence of a difference in sensitivity between the two laboratories (*P* < 0.001) (Table 1).

**Table 1:** Sensitivity, specificity, accuracy^a^, positive predictive value and negative predictive value of qPCR on pooled samples as compared to individual sample-based qPCR, for each one of the soil-transmitted helminth-specific qPCR assays, for both laboratories: Natural History Museum (NHM) and Smith College (SC)

Species	No. of pools tested		Sensitivity (95% CI)			Specificity (95% CI)			Accuracy (95% CI)			PPV (95% CI)		NPV (95% CI)	
	NHM	SC	NHM	SC	*P* ^b^	NHM	SC	*P* ^b^	NHM	SC	*P* ^b^	NHM	SC	NHM	SC
*N. americanus* ^c^	66	–	0.71 (0.52–0.86)	–	< 0.001	1.00 (0.90–1.00)	–	1	0.86 (0.84–0.89)	–	0.01	1.00 (0.85–1.00)	–	0.80 (0.65–0.90)	–
*A. lumbricoides*	72	56	0.69 (0.48–0.86)	1.00 (0.91–1.00)	< 0.001	1.00 (0.92–1.00)	1.00 (0.79–1.00)	1	0.89 (0.87–0.91)	1.00 (1.00–1.00)	0.03	1.00 (0.81–1.00)	1.00 (0.91–1.00)	0.85 (0.73–0.93)	1.00 (0.79–1.00)
*T. trichiura*	88	56	0.65 (0.50–0.79)	0.80 (0.64–0.91)	0.13	0.98 (0.87–1.00)	1.00 (0.79–1.00)	0.76	0.81 (0.77–0.84)	0.80 (0.75–0.85)	0.93	0.97 (0.83–1.00)	1.00 (0.89–1.00)	0.72 (0.58–0.83)	0.67 (0.45–0.84)
*A. ceylanicum*	60	36	0.82 (0.60–0.95)	1.00 (0.79–1.00)	0.07	0.95 (0.82–0.99)	1.00 (0.83–1.00)	0.64	0.90 (0.88–0.92)	1.00 (1.00–1.00)	0.14	0.90 (0.68–0.99)	1.00 (0.79–1.00)	0.90 (0.76–0.97)	1.00 (0.83–1.00)
*S. stercoralis*	60	36	0.70 (0.35–0.93)	1.00 (0.40–1.00)	0.22	0.96 (0.86–1.00)	0.81 (0.64–0.93)	0.03	0.92 (0.90–0.94)	0.70 (0.61–0.79)	0.01	0.78 (0.40–0.97)	0.40 (0.12–0.74)	0.94 (0.84–0.99)	1.00 (0.87–1.00)

^a^The accuracy of the pooling method was also calculated using the following formula: (true positives + true negatives)/number of pools, showing the proportion of the time when the pooled sample matches the result of the individual samples

^b^*P*-value calculated *via* chi-square test

^c^’Seeding’ experiment results (available only at SC): 44 pools tested; Sensitivity (95% CI): 1.00 (0.91–1.00); Specificity (95% CI): 1.00 (1.00–1.00); Accuracy (95% CI): 1.00 (1.00–1.00); PPV (95% CI): 1.00 (0.91–1.00); NPV (95% CI): 1.00 (0.40–1.00)

*Abbreviations*: PPV, positive predictive value; NPV, negative predictive value; CI, confidence interval

Estimates of specificity were consistently higher than those for sensitivity, suggesting the pooling approach has a low rate of false positives. Both *N. americanus* and *A. lumbricoides* had perfect specificity from NHM (1.00, 95% CI: 0.90–1.00 and 1.00, 95% CI: 0.92–1.00, respectively), whilst the same was true for *An. ceylanicum*, *A. lumbricoides* and *T. trichiura* at SC. All other estimates from both laboratories were above 0.90 except for *S. stercoralis* at SC (0.81, 95% CI: 0.64–0.93). There was no evidence of a difference in specificity estimates between NHM and SC for *A. lumbricoides* (*P* = 1.00), *T. trichiura* (*P* = 0.76) or *An. ceylanicum* (*P* = 0.64), but there was strong evidence of a difference for *S. stercoralis* (*P* = 0.03) (Table 1).

PPV estimates were generally high across all samples, with each species’ estimate of at least 0.90. The only exception was *S. stercoralis* with a PPV estimate of 0.78 (95% CI: 0.40–0.97) for NHM and 0.40 (95% CI: 0.12–0.74) at SC. NPV estimates showed much greater variability, especially from the NHM testing. Here, estimates ranged from 0.72 (95% CI: 0.58–0.83) for *T. trichiura* to 0.94 (95% CI: 0.84–0.99) for *S. stercoralis* (Table 1).

Pearson’s correlation coefficient (*r*) values between the individual aliquot qPCR results and the pooled qPCR results were generally consistent for the NHM and SC samples for each species with strong, positive correlations obtained from the *A. lumbricoides* samples (NHM: *r* = 0.75, *P* < 0.001; SC: *r* = 0.86, *P* < 0.001) and the *An. ceylanicum* samples (NHM: *r* = 0.93, *P* < 0.001; SC: *r* = 0.92, *P* < 0.001). The one exception was with regards to *S. stercoralis*, for which a strong positive correlation was identified for the NHM samples (*r* = 0.97, *P* < 0.001) but a very weak, and statistically insignificant, negative correlation was identified from the SC samples (*r* = − 0.07, *P* = 0.93) (Table 2).

**Table 2:** Pearson’s correlation values between individual and pooled qPCR-results (*P*-value) at both Smith College (SC) and Natural History Museum (NHM)

Species	No. of positive samples correctly identified through pooling Field samples		Pearson’s *r*-value (*P-*value) Field samples	
	NHM	SC	NHM	SC
*N. americanus* ^a^	22		0.86 (*P* < 0.001)	–
*A. lumbricoides*	18	40	0.75 (*P* < 0.001)	0.86 (*P* < 0.001)
*T. trichiura*	30	32	0.41 (*P* = 0.025)	0.32 (*P* = 0.070)
*An. ceylanicum*	18	16	0.93 (*P* < 0.001)	0.92 (*P* < 0.001)
*S. stercoralis*	7	4	0.97 (*P* < 0.001)	− 0.07 (*P* = 0.926)

^a^‘Seeding’ experiment results (available only at SC): 40 pools tested and identified as positive, Pearson’s *r*-value -0.26 (*P* = 0.112)

For the NHM samples, agreement in qPCR findings between both the 5-sample pool replicates and the A/B individual aliquots was moderate to high for all species, with Cohen’s kappa ranging from 0.66 to 1.00. Similarly, with the SC samples, *A. lumbricoides* and *An. ceylanicum* showed perfect agreement for both aliquots and 5-sample pools, whilst a strong agreement was found for *T. trichiura* 5-sample pool results. However, only weak evidence of agreement occurring more often than would be expected by chance was identified for the 5-sample pools for *S. stercoralis* (k = 0.44, *P* = 0.07) (Table 3).

**Table 3:** Degree of agreement in qPCR findings for all species of soil-transmitted helminths between A and B aliquots (for individual samples) and 1 and 2 samples (for pools) from Natural History Museum (NHM) and Smith College (SC) as calculated through Cohenʼs kappa statistic

Species	Method	Field samples				Seeding	
		NHM	*P*-value	SC	*P*-value	SC	*P-*value
*N. americanus*	Individual	0.67 (0.41–0.94)	< 0.001	–	–	1.00 (1.00–1.00)	< 0.001
	Pool	0.87 (0.69–1.00)	< 0.001	–	–	1.00 (1.00–1.00)	< 0.001
*A. lumbricoides*	Individual	0.67 (0.41–0.94)	< 0.001	1.00 (1.00–1.00)	< 0.001	–	–
	Pool	0.87 (0.69–1.00)	< 0.001	1.00 (1.00–1.00)	< 0.001	–	–
*T. trichiura*	Individual	1.00 (1.00–1.00)	< 0.001	1.00 (1.00–1.00)	< 0.001	–	–
	Pool	0.66 (0.43–0.89)	< 0.001	0.71 (0.45–0.97)	< 0.001	–	–
*An. ceylanicum*	Individual	1.00 (1.00–1.00)	< 0.001	1.00 (1.00–1.00)	< 0.001	–	–
	Pool	0.70 (0.43–0.97)	< 0.001	1.00 (1.00–1.00)	< 0.001	–	–
*S. stercoralis*	Individual	0.79 (0.40–1.00)	< 0.001	1.00 (1.00–1.00)	< 0.001	–	–
	Pool	0.87 (0.62–1.00)	< 0.001	0.44 (− 0.01–0.91)	0.07	–	–

Lastly, for all species, Cohen’s kappa found a very strong degree of agreement in qPCR findings (translated as positivity for that particular target) between the isolates originally obtained at SC and the pools subsequently created at NHM (k ≥ 0.77, *P* < 0.001) except for *N. americanus*, where a slightly weaker degree of agreement was identified (k = 0.51, *P* = 0.02) (Table 4). The raw numbers used for the analyses (number of true/false positives/negatives per set of pools) are provided in Additional file 1: Table S1.

**Table 4:** Degree of agreement in qPCR findings for all helminths tested between Smith College (SC) isolates and Natural History Museum (NHM) pools as calculated through Cohen’s kappa statistic

Species	No. of pools	Cohen’s kappa (95% CI)	*P*-value
*N. americanus*	17	0.51 (0.10–0.93)	0.02
*A. lumbricoides*	18	0.77 (0.48–1.00)	< 0.001
*T. trichiura*	22	1.00 (1.00–1.00)	< 0.001
*An. ceylanicum*	15	0.86 (0.59–1.00)	< 0.001
*S. stercoralis*	15	0.84 (0.55–1.00)	< 0.001

### Cost exercise

**Fig. 3:**
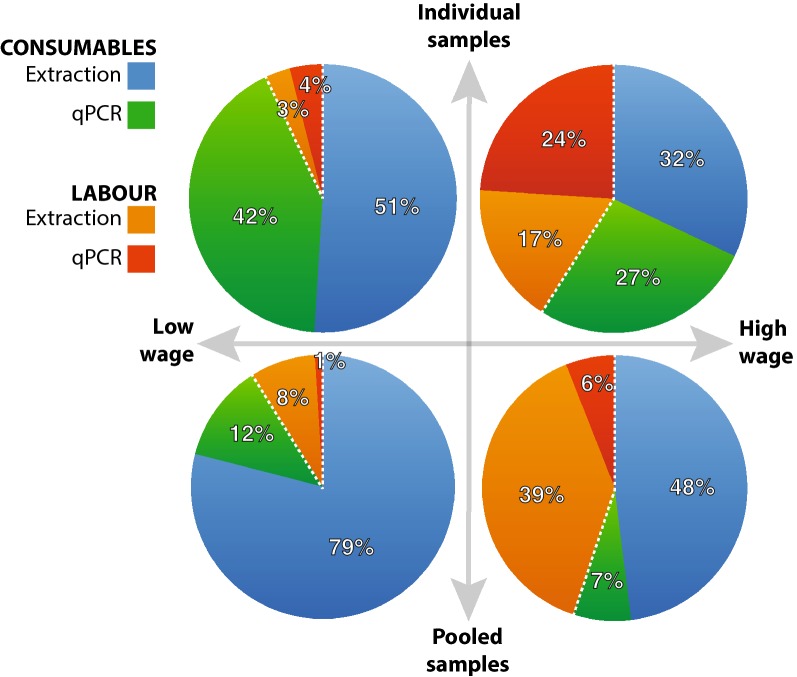
Cost analysis comparing individual *vs* pooled samples in both low- and high-wage settings where all samples are known to be negative for all the soil-transmitted helminth species of interest. Dashed white line separates consumable (extraction and qPCR reagents) from labour costs

**Fig. 4:**
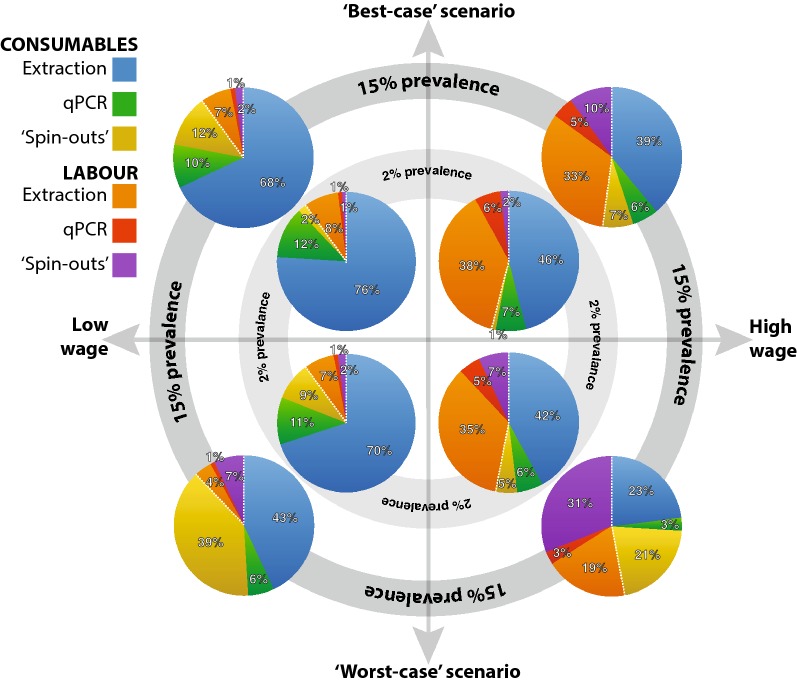
Cost analysis on pooling in both low- and high-wage settings in two different scenarios and for two levels of prevalence (2% and 15%) for a single soil-transmitted helminth species. Scenarios represent ‘best’ and ‘worst’ cases of positive sample distributions across 5-sample pools; see main text. Dashed white line separates consumable (extraction, qPCR and ‘spin-out’ reagents) from labour costs

In all graphs shown (Figs. 3 and 4) no absolute numbers are reported as this cost exercise would differ significantly based on income (wage), currency and technician competency which would affect labour time invested. Instead, we report relative proportions of total cost.

#### Simplest scenario: all samples are negative for the STH to be screened

In the simplest case where all the individual samples are negative (and thus, so are the pools), there is no need for ‘spin outs’; hence, only costs for labour and consumables (based on list prices online) are included (Fig. 3). In both low-income and high-income settings, labour is a slightly more expensive element than the consumables needed to process the samples in pools compared to the same samples processed individually (low-income setting: labour 9% and consumables 91% *versus* labour 7% and consumables 93%, high-income setting: labour 41% and consumables 59% *versus* labour 45% and consumable 55%, respectively). So, when all the samples are negative—or expected to be—there is no significant cost-savings when a pooling strategy is implemented compared to processing all the samples individually.

#### More complicated scenarios: impact of prevalence and its distribution to the pools

In this cost exercise, two scenarios including STH prevalence rates were considered; 2% and 15% prevalence of a particular STH. Taking the example of 1000 samples and a prevalence of 2% or 15%, this would result in 20 and 150 positive samples, respectively. Out of those pools, in the ‘best-case’ scenario (Fig. 4), 4 and 30 positive pools would have to be revisited, for extraction and processing. However, for the same number of samples and under the same prevalence rates, the ‘worst-case’ scenario would require 20 and 150 pools to be processed, for 2% and 15% prevalence respectively.

In Fig. 4, for the positive pools alone, the additional cost for labour and consumables needed for the ‘spin-outs’ was also estimated and incorporated to the graphs. In the ‘worst-case’ scenario, as the prevalence increases the labour cost also increases in both low and high-income settings. In the ‘best-case’ scenario, for the same parameters (low to high prevalence) only for the low-income settings is the consumable cost slightly higher, whereas in the high-income settings the labour drivers are higher as the prevalence increases.

## Discussion

The strategy of pooling has been considered an attractive way of screening multiple samples simultaneously for a particular target/pathogen, both in research and veterinary settings, potentially lowering the cost of labour or consumables needed [4, 10–12, 18, 27]. At the SC laboratory, some preliminary work on screening ‘pools of 10’ was conducted, and even though no dramatic loss of sensitivity was observed, the practicality of the process was deemed more challenging due to lack of sufficient equipment. For this reason, and upon initial cost assessment of consumable and reagent costs involved in ‘pooling’, we focused on assessing a strategy of using 5-sample pools.

The main query of this study was whether pooling is an appropriate strategy for the qualitative detection of STHs in a post-treatment population, where most individuals are expected to be identified as ‘negative’ (based on the diagnostic test chosen). In a setting with most samples being negative, most pooled samples will also be negative thus, potentially, reducing labour and consumable costs and the lower likelihood of having to re-examine individual samples when pools are found to be positive. Moreover, we aimed to show that pooling does not dramatically reduce the chances of the target detection by PCR (given the fact that it is further diluted as part of the pool). These questions are widely relevant for both veterinary [10] and clinical trials and epidemiological studies where large numbers of infected stool samples must be processed to assess infection presence and intensity [15, 26]. Our study focused on a qualitative assessment of the infection levels (presence/absence). The correlation of eggs found in a stool sample to worm burden and subsequently to intensity of infection is of paramount importance in epidemiological studies. A recent review of Papaiakovou et al. [34], addresses the concerns around quantitation of qPCR outputs and their subsequent correlation to egg numbers and, therefore, intensity of infection with confidence. We believe that qPCR has yet to achieve its potential for quantitative purposes given the limitations of PCR target selected, cell numbers present in eggs, and extraction efficiency. Additionally, the dilution of target through pooling will further hinder such quantitation. Thus, we decided to assess presence/absence of the target in both individuals and pools.

Our main objectives were to evaluate the successful formation of the pool, the potential for single sampling of the pool (to avoid reagent and labour cost inflation due to multiple sampling) and the subsequent detection of the diluted target with precision and accuracy. To our knowledge, this is the first time such queries have been interrogated to assist in strategic planning.

### Method development

Given prior research on the need to blend stool samples sufficiently [35], and the importance of STH egg disruption by utilizing a high-speed bead-based homogenizer [36–38] we acknowledged that any method developed to form pools would be critical, and the subsequent accurate detection of the evenly distributed targets upon dilution in the pool, would be challenging.

The development of a ‘pooling’ protocol that overcomes known limitations and meets all of the aforementioned expectations was relatively trivial for the ‘seeding experiment’, where only *N. americanus* eggs were recruited and tested. However, mixing or stirring the faecal pool with a sterile loop or low-power vortexer, was insufficient for the field-samples experiment, where the stool samples being recruited were positive for additional STH helminth species. The different consistencies of the stool samples involved, along with the low load of the infection in each one of the samples recruited, showed that adequate mixing was required. Furthermore, the need for both additional buffer and a bead-based beating step both to facilitate the homogeneous blending of the helminth eggs (or DNA) was also critical.

### Precision and reproducibility

A working protocol that showed overall statistically significant and acceptable agreement between individuals and pools (through kappa values) was developed. The protocol presented no apparent technical errors for any of the helminths tested. However, due to the complexity and hands-on time, the need to test protocol reproducibility between different technicians and laboratory settings also emerged. Sequentially, our study aimed to show that the protocol is duplicable by any adequately trained and competent technician. Hence, the same pooling workflow (Fig. 2) was compared at two different laboratories (SC and NHM).

Utilizing the pooling strategy as described herein, a generally low rate of false negatives is expected. Also, specificity does not seem to be an issue overall but of interest remains the lower PPV for the *S. stercoralis* which is discussed in a separate section below.

Last but not least, the list of samples chosen to be pooled had originally been extracted and tested at SC (using the same extraction protocol and the same qPCR assays). Aliquots from the same stool samples were selected to be extracted independently (individually, and as part of pools) at NHM. Almost absolute agreement was shown between individual samples originally and independently tested with qPCR at SC with the results (individual and pool) obtained from NHM.

### Single replicates *versus* duplicates

The Kappa estimates, comparing both individual aliquots and the pooled aliquots, showed a high degree of agreement, which suggests conducting the test twice may be unnecessary. For all species, agreement between 1 and 2 pool replicates was moderate to high for both laboratories. This provides strong statistical evidence that there is little need for multiple sampling. When processing large numbers of samples, the need for rapid and simple detection of the infection by single sampling is important due to the costs involved (reagents and labour). Using our developed protocol, with sufficient mixing and homogenization, there is clearly no need for multiple sampling (A and B in individuals, 1 and 2 in pools), since the infection/target seems to be evenly distributed following the workflow presented here.

For direct comparison of the individual samples forming the pool with the 5-sample pools *per se*, the individual samples constituting a pool were tested in duplicate, each pool was formed twice and the DNA from each pool was also extracted twice. Our study/protocol demonstrates that a thorough homogenization is critical for even distribution of the target present in stool samples. In that way, there is no reason or need for extracting DNA from the same sample/pool twice, and even in its most demanding format the protocol can be learned, implemented and reproducibly performed by suitably skilled technicians, as suggested by kappa values. Given the overall high degree of agreement, a conclusion that a single pool per 5 samples would be sufficient, can also be made.

### Paradoxes

Even though the specificity for *S. stercoralis* was not significantly different at SC compared to NHM, the PPV was slightly lower (individual samples identified as negatives when screened by PCR were deemed positive for *S. stercoralis* as part of the pools). However, this can be attributed to the lower prevalence of *S. stercoralis* in the SC samples (10%) as compared to other parasites (at around 40–50%). As a worked example demonstrating the impact of prevalence on PPV, if the sensitivity and specificity for *S. stercoralis* calculated at SC remained constant (1.00 and 0.625, respectively) but prevalence was increased to 30%, the “new” PPV would be calculated as 0.79, i.e. more consistent with findings from NHM.

Moreover, the presence of larvae instead of eggs and the additional beating steps in the pool (*versus* individual samples), may have contributed to the infection being ‘missed’ at certain individual samples. It is suspected that further homogenization of larvae facilitated target detection in the pool, but not in the aliquot from the individual. Another possible explanation would be that ‘weak’ infections, unable to be detected in the individuals due to limits of detection of the qPCR assay, were collectively surpassing the detection threshold as part of the pool. All the individual samples had been previously screened independently, as mentioned earlier. Since all the samples previously reported as negatives were indeed negatives when tested in the laboratory, we are ruling out the chance of contamination than can lead to ‘false positive’ results. These samples were ‘true positive’ for *S. stercoralis*, hence we believe the respective pools were not ‘false positive’. However, a higher prevalence of *S. stercoralis* in a given dataset would be needed in order to draw any further conclusions.

In the case of *N. americanus* and *A. lumbricoides*, since there was almost perfect agreement between individuals and respective pools, the slightly weaker agreement between original extracts and aliquots run at NHM may indicate a lack of adequate homogenization in the original sample.

### Cost and time savings with pooling

The authors acknowledge that a viable and cost-effective protocol must not be too complicated or too laborious to set-up. Additionally, any protocol established as a time-saving strategy cannot be less cost-effective than processing the same number of samples individually. For this reason, a broad indicative cost analysis was carried out by our team. We calculated costs based on 1000 samples requiring processing; small enough for easy analysis, large enough to represent a case where pooling might be justified. For consistency and accurate reporting, the current protocol included all the extractions in duplicate and the formation and subsequent extraction of the same pool twice; these components were also part of the cost-model and comparison.

For every pool positive for a single parasite, there is the need to ‘re-visit’ the individual samples that originally formed the pool, repeat the extraction step for each component sample and test each extract for the parasite of interest. For every additional parasite detected in the pooled sample, the additional cost increase is translated to consumables and the time to perform qPCR. However, pooling in the presence of positives adds to the overall cost of this alternative strategy relative to single sample processing. However, there remains room for further optimization of the current workflow (larger capacity homogenizers, purification and liquid handling systems). With a streamlined protocol in place capable of eliminating ‘redundant’ steps (three *versus* two rounds of homogenization for the pool) further simplifying the protocol may be possible, providing additional time and cost savings even when low percentages of STH prevalence are expected. Also, in cases where microscopy data may be available for individual samples, a ‘strategic pooling’ approach could be to use the samples identified as negatives for forming the pools and process the rest individually.

We acknowledge that our cost estimates based on list prices might not accurately reflect potential cost-saving with bulk or similar discounted purchasing, but the relative costs are likely indicative of broader trends. In our cost exercise, we included a simple case, where all samples are expected to be negative and a more complicated case with the infection present in a population. In the latter, we included only a ‘worst-’ and a ‘best-case’ scenario, along with only two levels of prevalence (2% and 15%) for a single STH species, based on low- and high-income countries. We understand that a realistic situation of the prevalence and distribution of any helminth present will lie somewhere in between. A more comprehensive mathematical cost model will include coefficients such as prevalence rates for a single STH species or more, cost from ‘spin-outs’ of ‘false positives’ or ‘penalty’ of false negatives in the long term, along with tailored wages to suggest a few.

### To pool or not to pool

The main drive for developing and testing a pooling protocol has always been the potential savings in labour and consumables, but the additional dilution of the target and subsequent loss of sensitivity of the diagnostic method employed, has been of major concern. Recent research has challenged and augmented those concerns; pooling, might not be the cost-effective technique once hoped for.

Logistical and operational costs [18], special equipment or additional consumables needed (this study), the necessity of reproducibility (this study) and single-sample granularity in the infection present (revealing the ‘positive’ individuals that contribute to a ‘positive’ pool; this study), or generally prevalence in a given population [21], labour cost and study size are amongst the pivotal factors that will determine whether a pooling protocol will actually be beneficial and worthwhile.

## Conclusions

We describe a successful pooling strategy that lessens the presence of false negative results, demonstrates reproducibility and minimizes the need for multiple replicates as long as there is sufficient mixing in the individual stools forming the pool. Such a methodology is yet to be simplified and tailored to the needs of any interventions. Even though pooling is more likely a better fit for low STH prevalence or surveillance areas and clusters where interruption of transmission is approached (< 2%), the findings and approach of this study will facilitate future protocol developments and optimizations. Our hope is that this study will assist in decision-making on single versus pooling implementation when considering end-to-end processes, budgeting and time considerations in diagnosing STH in faecal samples.

## Supplementary information

**Additional file 1: Table S1.** Raw numbers of true positives/negatives for both ‛seedingʼ experiment (Smith College) using *N. americanus* egg counts and field-sample testing (Smith College and Natural History Museum) for both individual samples and pools.

## Data Availability

The datasets used and/or analysed during the present study are available from the corresponding author upon reasonable request.

## Abbreviations

STH: soil-transmitted helminths
qPCR: quantitative polymerase chain reaction
SC: Smith College
NHM: Natural History Museum

## References

[1] Wahlquist SP, Williams RM, Bishop H, Addiss DG, Stewart JM, Finton RJ (1991). Use of pooled formalin-preserved fecal specimens to detect *Giardia lamblia*. J Clin Microbiol..

[2] Singer RS, Cooke CL, Maddox CW, Isaacson RE, Wallace RL (2006). Use of pooled samples for the detection of *Salmonella* in feces by polymerase chain reaction. J Vet Diagn Investig.

[3] Mitchell S, Pagano M (2012). Pooled testing for effective estimation of the prevalence of *Schistosoma mansoni*. Am J Trop Med Hyg..

[4] Mekonnen Z, Meka S, Ayana M, Bogers J, Vercruysse J, Levecke B (2013). Comparison of individual and pooled stool samples for the assessment of soil-transmitted helminth infection intensity and drug efficacy. PLoS Negl Trop Dis..

[5] Vlaminck J, Cools P, Albonico M, Ame S, Ayana M, Bethony J (2018). Comprehensive evaluation of stool-based diagnostic methods and benzimidazole resistance markers to assess drug efficacy and detect the emergence of anthelmintic resistance: a Starworms study protocol. PLoS Negl Trop Dis..

[6] Shipitsyna E, Shalepo K, Savicheva A, Unemo M, Domeika M (2007). Pooling samples: the key to sensitive, specific and cost-effective genetic diagnosis of *Chlamydia trachomatis* in low-resource countries. Acta Derm Venereol.

[7] Lo NC, Coulibaly JT, Bendavid E, N’Goran EK, Utzinger J, Keiser J (2016). Evaluation of a urine pooling strategy for the rapid and cost-efficient prevalence classification of schistosomiasis. PLoS Negl Trop Dis..

[8] Verstraeten T, Farah B, Duchateau L, Matu R (1998). Pooling sera to reduce the cost of HIV surveillance: a feasibility study in a rural Kenyan district. Trop Med Int Health..

[9] Zaky WI, Tomaino FR, Pilotte N, Laney SJ, Williams SA (2018). Backpack PCR: a point-of-collection diagnostic platform for the rapid detection of *Brugia* parasites in mosquitoes. PLoS Negl Trop Dis..

[10] Eysker M, Bakker J, van den Berg M, van Doorn DCK, Ploeger HW (2008). The use of age-clustered pooled faecal samples for monitoring worm control in horses. Vet Parasitol..

[11] Pedersen KS, Johansen M, Jorsal SE, Nielsen JP, Bækbo P, Angen Ø (2014). Pooling of porcine fecal samples for quantification of *Lawsonia intracellularis* by real-time polymerase chain reaction. J Vet Diagn Investig.

[12] Clasen J, Mellerup A, Olsen JE, Angen Ø, Folkesson A, Halasa T (2016). Determining the optimal number of individual samples to pool for quantification of average herd levels of antimicrobial resistance genes in Danish pig herds using high-throughput qPCR. Vet Microbiol..

[13] Kenyon F, Rinaldi L, McBean D, Pepe P, Bosco A, Melville L (2016). Pooling sheep faecal samples for the assessment of anthelmintic drug efficacy using McMaster and Mini-FLOTAC in gastrointestinal strongyle and *Nematodirus* infection. Vet Parasitol..

[14] Anderson R, Farrell S, Turner H, Walson J, Donnelly CA, Truscott J (2017). Assessing the interruption of the transmission of human helminths with mass drug administration alone: optimizing the design of cluster randomized trials. Parasites Vectors..

[15] Ásbjörnsdóttir KH, Ajjampur SSR, Anderson RM, Bailey R, Gardiner I, Halliday KE (2018). Assessing the feasibility of interrupting the transmission of soil-transmitted helminths through mass drug administration: the DeWorm3 cluster randomized trial protocol. PLoS Negl Trop Dis..

[16] Pullan RL, Smith JL, Jasrasaria R, Brooker SJ (2014). Global numbers of infection and disease burden of soil transmitted helminth infections in 2010. Parasites Vectors..

[17] Kure A, Mekonnen Z, Dana D, Bajiro M, Ayana M, Vercruysse J (2015). Comparison of individual and pooled stool samples for the assessment of intensity of *Schistosoma mansoni* and soil-transmitted helminth infections using the Kato-Katz technique. Parasites Vectors..

[18] Leta GT, French M, Dorny P, Vercruysse J, Levecke B (2018). Comparison of individual and pooled diagnostic examination strategies during the national mapping of soil-transmitted helminths and *Schistosoma mansoni* in Ethiopia. PLoS Negl Trop Dis..

[19] Medley GF, Turner HC, Baggaley RF, Holland C, Hollingsworth TD (2016). The role of more sensitive helminth diagnostics in mass drug administration campaigns: elimination and health impacts. Adv Parasitol..

[20] Khurana S, Sethi S (2017). Laboratory diagnosis of soil transmitted helminthiasis. Trop Parasitol..

[21] Truscott JE, Dunn JC, Papaiakovou M, Schaer F, Werkman M, Littlewood DT (2019). Calculating the prevalence of soil-transmitted helminth infection through pooling of stool samples: choosing and optimizing the pooling strategy. PLoS Negl Trop Dis..

[22] Pilotte N, Papaiakovou M, Grant JR, Bierwert LA, Llewellyn S, McCarthy JS (2016). Improved PCR-based detection of soil-transmitted helminth infections using a next-generation sequencing approach to assay design. PLoS Negl Trop Dis..

[23] Papaiakovou M, Pilotte N, Grant JR, Traub RJ, Llewellyn S, McCarthy JS (2017). A novel, species-specific, real-time PCR assay for the detection of the emerging zoonotic parasite *Ancylostoma ceylanicum* in human stool. PLoS Negl Trop Dis..

[24] Lim MD, Brooker SJ, Belizario VY, Gay-Andrieu F, Gilleard J, Levecke B (2018). Diagnostic tools for soil-transmitted helminths control and elimination programs: a pathway for diagnostic product development. PLoS Negl Trop Dis..

[25] Becker SL, Liwanag HJ, Snyder JS, Akogun O, Belizario V, Freeman MC (2018). Toward the 2020 goal of soil-transmitted helminthiasis control and elimination. PLoS Negl Trop Dis..

[26] Tabi ES, Eyong EM, Akum EA, Löve J, Cumber SN (2018). Soil-transmitted helminth infection in the Tiko Health District, South West Region of Cameroon: a post-intervention survey on prevalence and intensity of infection among primary school children. Pan Afr Med J..

[27] Muñoz-Zanzi C, Thurmond M, Hietala S, Johnson W (2006). Factors affecting sensitivity and specificity of pooled-sample testing for diagnosis of low prevalence infections. Prev Vet Med..

[28] WHO Expert Committee on the Control of Schistosomiasis (2001: Geneva, Switzerland) & World Health Organization. Prevention and control of schistosomiasis and soil-transmitted helminthiasis: report of a WHO expert committee. Geneva: World Health Organization; 2002. https://apps.who.int/iris/handle/10665/42588; Accessed 23 Apr 2019. 12592987

[29] Papaiakovou M, Pilotte N, Baumer B, Grant J, Asbjornsdottir K, Schaer F (2018). A comparative analysis of preservation techniques for the optimal molecular detection of hookworm DNA in a human fecal specimen. PLoS Negl Trop Dis..

[30] Papaiakovou M, Pilotte N, Hu Y, Aroian RV, Walson JL, Williams SA (2017). Pool the stool: pooling stool samples as a strategy as a strategy for increasing the efficiency and effectiveness of real-time PCR for soil-transmitted helminths (STH). Am Soc Trop Med Hyg..

[31] Ercumen A, Benjamin-Chung J, Arnold BF, Lin A, Hubbard AE, Stewart C, et al. Effects of water, sanitation, handwashing and nutritional interventions on soil-transmitted helminth infections in young children: a cluster-randomized controlled trial in rural Bangladesh. bioRxiv. 2019;512509. 10.1371/journal.pntd.0007323 PMC6519840 31050672

[32] Clopper CJ, Pearson ES (1934). The use of confidence or fiducial limits illustrated n the case of the bionomial. Biometrika..

[33] Watson PF, Petrie A (2010). Method agreement analysis: a review of correct methodology. Theriogenology..

[34] Papaiakovou M, Gasser RB, Littlewood DTJ (2019). Quantitative PCR-based diagnosis of soil-transmitted helminth infections: faecal or fickle?. Trends Parasitol..

[35] Krauth SJ, Coulibaly JT, Knopp S, Traoré M, NʼGoran EK, Utzinger J (2012). An in-depth analysis of a piece of shit: distribution of *Schistosoma mansoni* and hookworm eggs in human stool. PLoS Negl Trop Dis..

[36] Andersen UV, Haakansson IT, Roust T, Rhod M, Baptiste KE, Nielsen MK (2013). Developmental stage of strongyle eggs affects the outcome variations of real-time PCR analysis. Vet Parasitol..

[37] Demeler J, Ramünke S, Wolken S, Ianiello D, Rinaldi L, Gahutu JB (2013). Discrimination of gastrointestinal nematode eggs from crude fecal egg preparations by inhibitor-resistant conventional and real-time PCR. PLoS ONE.

[38] Espírito-Santo MCC, Alvarado-Mora MV, Pinto PLS, Carrilho FJ, Pinho JRR, Gryschek RCB (2012). Two sequential PCR amplifications for detection of *Schistosoma mansoni* in stool samples with low parasite load. Rev Inst Med Trop Sao Paulo..

